# Prognostic Implications of Serum Lipid Metabolism over Time during Sepsis

**DOI:** 10.1155/2015/789298

**Published:** 2015-08-17

**Authors:** Sang Hoon Lee, Moo Suk Park, Byung Hoon Park, Won Jai Jung, In Seon Lee, Song Yee Kim, Eun Young Kim, Ji Ye Jung, Young Ae Kang, Young Sam Kim, Se Kyu Kim, Joon Chang, Kyung Soo Chung

**Affiliations:** ^1^Division of Pulmonary and Critical Care Medicine, Department of Internal Medicine, Institute of Chest Diseases, Severance Hospital, Yonsei University College of Medicine, 50-1 Yonsei-ro, Seodaemun-gu, Seoul 120-752, Republic of Korea; ^2^Division of Respiratory and Critical Care Medicine, Department of Internal Medicine, Korea University Anam Hospital, Korea University College of Medicine, 73 Inchon-ro, Seongbuk-gu, Seoul 136-705, Republic of Korea

## Abstract

*Background*. Despite extensive research and an improved standard of care, sepsis remains a disorder with a high mortality rate. Sepsis is accompanied by severe metabolic alterations. *Methods*. We evaluated 117 patients with sepsis (severe sepsis [*n* = 19] and septic shock [*n* = 98]) who were admitted to the intensive care unit. Serum cholesterol, triglyceride (TG), high-density lipoprotein (HDL), low-density lipoprotein (LDL), free fatty acid (FFA), and apolipoprotein (Apo) A-I levels were measured on days 0, 1, 3, and 7. *Results*. Nonsurvivors had low levels of cholesterol, TG, HDL, LDL, and Apo A-I on days 0, 1, 3, and 7. In a linear mixed model analysis, the variations in TG, LDL, FFA, and Apo A-I levels over time differed significantly between the groups (*p* = 0.043, *p* = 0.020, *p* = 0.005, and *p* = 0.015, resp.). According to multivariate analysis, TG levels and SOFA scores were associated with mortality on days 0 and 1 (*p* = 0.018 and *p* = 0.008, resp.). *Conclusions*. Our study illustrated that TG levels are associated with mortality in patients with sepsis. This may be attributable to alterations in serum lipid metabolism during sepsis, thus modulating the host response to inflammation in critically ill patients.

## 1. Introduction

Although mortality caused by severe sepsis with or without shock decreased between 2000 and 2012, it remains a leading cause of inhospital death [1]. There is no proven targeted therapy for severe sepsis, despite intensive research and randomized controlled trials. The total hospital costs associated with sepsis syndrome are increasing annually [2]. Furthermore, the incidence of sepsis has increased worldwide [3].

Lipopolysaccharide (LPS), the major component of the outer membrane of Gram-negative bacteria, plays a key role in the initiation of sepsis. After LPS binds to CD14, the inflammatory cascade begins [4, 5]. In addition, there is a significant transition in the distribution of circulating lipoproteins before and after sepsis [6]. Previous studies illustrated that lipoprotein plays an important role in LPS binding and neutralization, enzyme incorporation, including paraoxonase and platelet-activating factor acetylhydrolase, inhibition of the expression of endothelial cell adhesion, and stimulation of the expression of endothelial nitric oxide synthase in vitro [7, 8].

Cholesterol and lipoprotein levels change rapidly over time in patients with proinflammatory conditions, especially in critically ill intensive care unit (ICU) patients with severe infection or sepsis [8, 9]. Patients with severe sepsis have low levels of cholesterol, including high-density lipoprotein (HDL), low-density lipoprotein (LDL), and apolipoprotein A-I (Apo A-I) and high levels of triglycerides (TGs) and free fatty acids (FFAs) [9, 10].

Although lipid changes over time have been studied previously, only HDL has been studied extensively. There are only a few studies examining changes in serum lipid levels other than HDL over time in patients with sepsis, and the prognostic implications of changes in lipoprotein content remain unclear. Furthermore, most of these studies of lipid profiles were conducted in Western populations. Recently, simvastatin and rosuvastatin failed to provide better clinical outcomes in patients with acute respiratory distress syndrome (ARDS) or sepsis-associated ARDS [11, 12]. As a result, there are controversies related to the concentration of lipids [4, 13, 14], and further studies with Asian patients are needed because of ethnic differences, namely, low body mass indices (BMIs) and low-fat diets. To the best of our knowledge, no prior studies examined lipid profiles and sepsis using multivariate and survival analyses. The purpose of this study was to examine the difference and dynamic changes in serum lipid levels, including Apo A-I, in ICU patients with sepsis and evaluate whether these lipids are associated with prognosis.

## 2. Methods

### 2.1. Study Subjects

**Figure 1:**
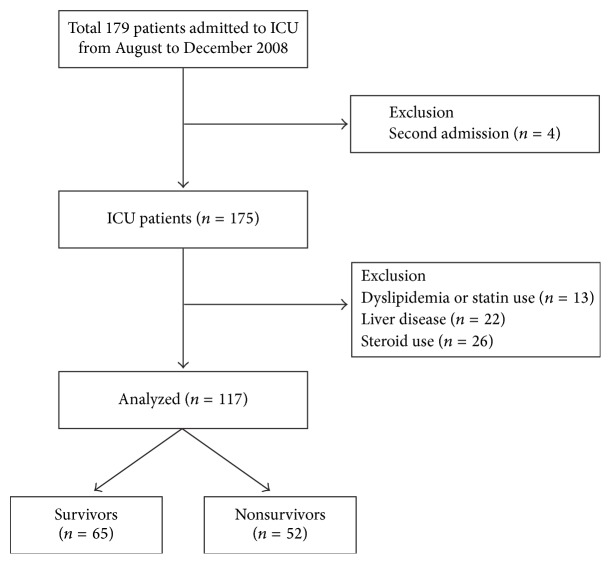
Flow chart of the inclusion and exclusion of patients in the study. A total of 179 patients were enrolled between August and December 2008, and 117 patients were included in the analysis. Patients who were being admitted for a second time (*n* = 4), those using steroids (*n* = 26), and those with a history of dyslipidemia (*n* = 13) or liver disease (*n* = 22) were excluded. Note: one patient had dyslipidemia and liver disease and two patients had liver disease and used steroids (≥15 mg/day) within the previous 7 days.

This was a prospective blood sampling and retrospective data analysis of a cohort of patients admitted to the medical ICU at a 2500-bed tertiary university medical center in Seoul, South Korea. The medical ICU is a 30-bed closed unit that is managed by certified ICU care specialists who only care for ICU patients. We enrolled patients admitted to the ICU with sepsis between August and December 2008; a total of 179 ICU patients were enrolled. Patients were excluded if they met specific criteria: age less than 18 years, pregnancy, ICU readmission, and a need for primary cardiac care. Patients with liver disease (hepatitis B, hepatitis C, immune hepatitis, liver cirrhosis, and hepatocellular carcinoma), dyslipidemia, or a history of statin or steroid (≥15 mg/day) use within the previous 7 days were also excluded from the study. Similarly, patients who had been treated with corticosteroid therapy for septic shock prior to admission to ICU were excluded. Finally, 117 patients were included for analysis in this study (Figure 1).

Sepsis was clinically defined as the identification of an infection site and three or four systemic inflammatory response syndrome (SIRS) criteria: (1) body temperature less than 36°C (96.8°F) or greater than 38°C (100.4°F); (2) heart rate greater than 90 beats/min; (3) tachypnea of greater than 20 breaths/min or an arterial partial pressure of carbon dioxide less than 4.3 kPa (32 mmHg); and (4) white blood cell count of less than 4000 cells/mm³ (4 × 10^9^ cells/L) or greater than 12,000 cells/mm³ (12 × 10^9^ cells/L) [15]. Septic shock was defined as the state of sepsis with persistent hypotension despite adequate volume resuscitation [16, 17]. All eligible patients in this study were managed according to “Surviving sepsis campaign: international guidelines for management of severe sepsis and septic shock: 2008” [18]. Once admitted to the ICU, patients were evaluated by a specialized nutrition team, who estimated the nutrition status of each patient and evaluated the possibility of enteral feeding. In total, 66 patients (56.4%) were supported by total parenteral nutrition, and 51 patients (43.6%) were supported by enteral feeding. Each patient was supplied with 20–30 kcal/kg/day of nutrition, depending on the patient's nutrition status, according to the guidelines of the American Society for Parenteral and Enteral Nutrition or the European Society for Clinical Nutrition and Metabolism [19, 20]. Additionally, patients were treated using an insulin protocol, targeting a level of 100–140 mg/dL, which we developed on the basis of a modification of the Yale protocol [21]. Propofol, which affects lipid profiles, was not used in this study.

For each patient, data on age, gender, BMI, comorbidity, infection strain, administration of vasoactive drugs, length of ICU stay, and 28-day mortality were collected. In addition, Acute Physiology and Chronic Health Evaluation II (APACHE II) scores, Sequential Organ Failure Assessment (SOFA) scores, and laboratory data, including cholesterol, TG, HDL, LDL, FFA, and Apo A-I serum levels, were collected on days 0 (ICU admission), 1, 3, and 7.

### 2.2. Blood Sampling and Assays

Blood samples were obtained using an indwelling arterial catheter or via venous puncture as soon as possible after the patient was admitted to the ICU. Blood samples from days 1, 3, and 7 were obtained at a fixed time (5 AM) for all eligible patients. The levels of total cholesterol (normal range: 100–220 mg/dL), HDL (normal range: 40–86 mg/dL), TG (normal range: 44–240 mg/dL), LDL (normal range: 70–169 mg/dL), and FFA (normal range: 150–600 *μ*Eq/L) were measured using commercial kits with an automated analyzer (Hitachi 7600-200-DDP, Hitachi Ltd., Tokyo, Japan). Apo A-I (normal range: 0.0–25.0 mg/dL) was measured using an autoanalyzer (Hitachi 7600-210 P-modulator, Hitachi Ltd.).

### 2.3. Statistical Analysis

Continuous variables were compared using Student's* t*-test or Mann-Whitney's *U* test, depending on the distribution, and were presented as the mean ± standard deviation or median (interquartile range). Categorical variables were compared using Pearson's chi-squared test or Fisher's exact test and were presented as absolute frequencies and percentages. The changes in serum levels in the lipid profile over time were compared between the groups using linear mixed model analysis after adjusting for age, sex, and BMI. The effect of age, gender, BMI, SOFA scores, and serum lipid levels on survival was evaluated using Cox proportional hazard models. Pearson's correlation was performed to investigate the association between lipid levels and SOFA scores. The predictive receiver operating characteristic (ROC) curves of 28-day mortality were compared between the SOFA score and SOFA score/TG level. The effects of TG levels on survival were assessed using Cox proportional hazards models. An adjusted *p* value <0.05 was considered statistically significant. All statistical analyses were performed using SPSS version 20 (SPSS, Chicago, IL, USA).

### 2.4. Ethics Statement

The study protocol was reviewed and approved by the Institutional Review Board (IRB) of Yonsei University Health Service, Severance Hospital (IRB approval number 4-2008-0099). Informed consent was obtained from patients or their next of kin.

## 3. Results

The baseline demographic and clinical characteristics of the study subjects (*n* = 117) at admission to the ICU (day 0) are shown in Table 1. The mean age of the 117 patients was 62.7 ± 16.2 years. Age, gender, and BMI were similar between the survivor and nonsurvivor groups. SOFA and APACHE II scores were elevated in the nonsurvivor group (*p* = 0.008 and *p* = 0.082, resp.). The lungs were the most commonly infected sites (80.3%). There were no significant differences between the two groups with respect to comorbidities. In addition, no significant differences were observed for clinical parameters. C-reactive protein (CRP) levels were elevated in the nonsurvivor group, albeit not significantly compared to the survivor group. Albumin levels were also not significantly different between the two groups. However, TG and FFA levels were significantly higher in the survivor group than in the nonsurvivor group. The levels of other lipoproteins (cholesterol, HDL, LDL, and Apo A-I) were also increased in the survivor group; however, the differences between the groups were not statistically significant.

**Figure 2:**
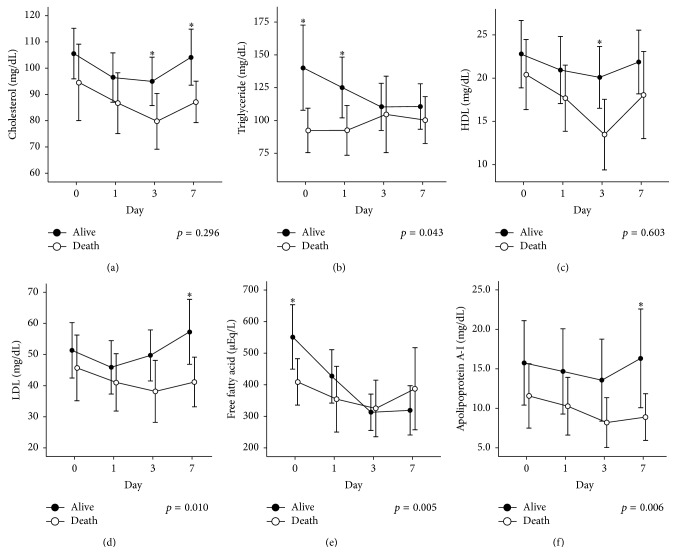
Time course of lipid levels in the survivor and nonsurvivor groups. Data for cholesterol (a), triglyceride (b), high-density lipoprotein (c), low-density lipoprotein (d), free fatty acid (e), and apolipoprotein A-I (f) are shown. Data were collected on the day of admission and days 1, 3, and 7 after admission. The circle and bar represent the mean value and standard error of the mean, respectively. Significant differences between the survivor and nonsurvivor groups are indicated by *∗*.

The changes in serum lipid levels over time are shown in Figure 2. Serum lipid and lipoprotein levels were investigated from the day of admission to the ICU (day 0) to day 7. Changes in TG, LDL, FFA, and Apo A-I levels over time differed significantly between the two groups (*p* = 0.043, *p* = 0.010, *p* = 0.005, and *p* = 0.006, resp.). However, changes in cholesterol and HDL levels over time did not differ significantly between the two groups. At different time points, there was a significant gap in the mean value for each lipid and lipoprotein between the two groups. Cholesterol levels were significantly elevated in the survivor group on days 3 and 7 compared to those of the nonsurvivor group. In addition, TG content was significantly elevated in the survivor group on days 0 and 1. Furthermore, significant differences were observed for HDL on day 3, LDL on day 7, FFA on day 0, and Apo A-I on day 7.

Using a Cox proportion hazard model, we performed survival analysis for each lipid level after adjusting for age, gender, BMI, and SOFA score on days 0, 1, 3, and 7 (Table 2). A total of 24 Cox proportion hazard models were constructed. On days 0 and 1, TG levels were significantly associated with survival. The hazard ratio (HR) decreased with TG levels (*p* = 0.015, HR = 0.991, and 95% confidence interval [CI] = 0.984–0.998 on day 0; *p* = 0.002, HR = 0.988, and 95% CI = 0.980–0.995 on day 1). Elevated LDL content on day 1 was associated with poor prognosis (*p* = 0.047, HR = 1.015, and 95% CI = 1.000–1.030). There was no significant association between the other lipids and survival.

**Figure 3:**
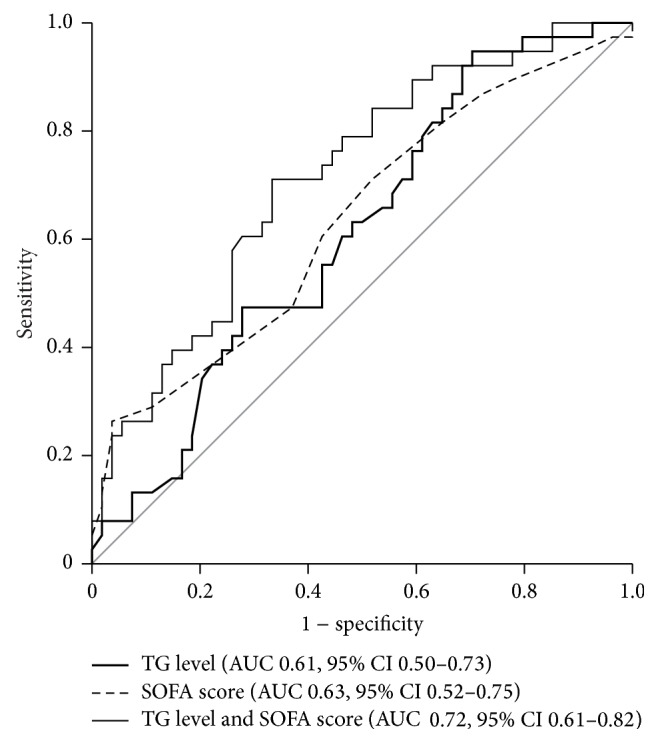
Receiver operator characteristic (ROC) curves of triglyceride levels and Sequential Organ Failure Assessment (SOFA) scores to predict mortality. On the day of admission, the area under curve for triglyceride levels was 0.62 (sensitivity = 94.7%, specificity = 29.6, and *p* = 0.066), and that for the SOFA score was 0.63 (sensitivity = 21.2%, specificity = 96.9, and *p* = 0.019). In addition, adjusting for triglyceride levels with the SOFA score significantly improved the predictive accuracy of 28-day mortality (AUC = 0.716, sensitivity = 71.1%, specificity = 66.7%, and *p* < 0.001).

The correlation between serum lipid levels and SOFA scores is shown in Table 3. Overall, the level of each lipid correlated well with the SOFA score, excluding TG and FFA. TG levels were not correlated with SOFA scores for the 7 days of ICU stay. The continuous TG level/SOFA score curve is shown in Figure 3, and it was a more accurate predictor of the 28-day mortality rate in ICU patients with sepsis than the SOFA score alone (area under the curve [AUC] = 0.717, *p* < 0.001 and AUC = 0.634, *p* = 0.029, resp.).

## 4. Discussion

The majority of studies performed previously to investigate sepsis focused on the role of HDL or Apo A-I and on the comparative analysis of mean values between survivor and nonsurvivor groups or between sepsis and severe sepsis groups [10, 22, 23]. In this study, we analyzed serum lipid levels over time and the association of lipid levels with survival in patients with sepsis.

Both HDL and Apo A-I were previously revealed to be potential therapeutic agents for sepsis. Guo et al. demonstrated that Apo A-I knockout mice were more susceptible to septic conditions and had a low capacity for LPS neutralization or clearance, corticosterone generation, and impaired leukocyte recruitment [24]. In addition, McDonald et al. reported that recombinant HDL (rHDL) reduced the severity of organ damage in rats preconditioned with* E. coli*. However, in their study, rats pretreated with rHDL did not display differences in hypotension events or serum levels of TNF-*α* [25]. Furthermore, Chenaud et al. illustrated that low serum levels of Apo A-I at ICU admission were related to SIRS exacerbation [10].

To date, only a few studies conducted survival analyses according to serum lipid levels in patients with sepsis. In our study, only TG levels on days 0 and 1 and LDL levels on day 1 were associated with survival. Although other lipids and lipoproteins illustrated significant differences over time between the survivor and nonsurvivor groups, no significant differences were found via survival analysis. This may be explained by the complexity of ICU patients with sepsis. In contrast to previous studies, which performed simple comparisons between survivors and nonsurvivors, our study reported that increased TG serum levels were significantly associated with a decreased mortality rate [4]. Harris et al. [26] and Barcia and Harris [6] explained this situation using the lipoprotein binding capacity of endotoxin and host defense mechanism to infection. The data suggested that the mean TG level for the survivor group in our study was similar to that in the low TG level group (survivor group) in other studies [10, 23]. Furthermore, Chiarla et al. reported that hypocholesterolemia, often paralleled by low TG levels, is associated with a poor prognosis [27]. Thus, both hypotriglyceridemia and hypertriglyceridemia could be related with poor outcomes in patients with sepsis. The absolute TG level may be important to some degree for the prediction of mortality. Choi et al. reported that both high and low TG levels were risk factors for poor early outcomes after acute ischemic stroke [28]. In addition, it is possible that racial and ethnic differences could have affected the results. In general, Korean diets are low in fat. Therefore, the Koreans usually have relatively low BMIs and low lipid blood levels compared to the Europeans and Americans. These ethnic features could explain the different results in our study in comparison to previous studies. Therefore, extremely low TG values may be associated with poor prognoses in ICU patients with sepsis. Similarly, the HDL levels in our study were lower than previously published values [10, 14, 22]. These relatively low HDL levels may explain why HDL levels did not differ significantly between the survivor and nonsurvivor groups. Recently, in two large, multicenter, double-blind, randomized, placebo-controlled studies, simvastatin and rosuvastatin did not improve clinical outcomes in patients with ARDS or sepsis-associated ARDS (28- or 60-day inhospital mortality, ventilator-free days, or days free of nonpulmonary organ failure) [11, 12]. These findings, however, disagree with previous studies [29–31]. As reported in these two large, randomized studies, our study reported that in contrast to other studies, in particular, hypertriglyceridemia is related with poor prognoses [4, 32]. Further large-scale studies are needed to investigate the therapeutic role of HDL and the changes in HDL levels over time in patients with sepsis.

We examined the correlation between the SOFA score and lipid levels (Table 3). We found that TG levels did not correlate well with the SOFA score, unlike the other lipids. Thus, we hypothesized that when TG levels are added to the SOFA score, 28-day mortality predictions would be more precise compared to using the SOFA score alone. The ROC curve using TG level/SOFA score on day 0 exhibited a wider AUC than the SOFA score alone, meaning that the 28-day mortality prediction was more accurate. Li et al. reported that the combination of soluble triggering receptor expression on myeloid cells-1 (sTREM-1), procalcitonin levels, and SOFA scores could be a powerful predictor for patients with sepsis [33]. Previously, serial measurements of lactate and procalcitonin were also helpful in the prediction of 28-day mortality [34]. In our study, TG, as well as the aforementioned biomarkers, was identified as a potential prognostic factor for patients with sepsis.

There are some limitations to the current study. First, we did not consider the nutritional status of the patients before ICU admission, and we did not accurately calculate caloric intake during the ICU care period. Weight history, physical examination (e.g., skin and mucous membranes), bowel function, and laboratory assessments, including prealbumin, retinol-binding protein, and transferrin levels, were not investigated, which could be important for the results of this study. However, a specialized nutrition team managed the ICU patients with personalized nutrition assessments using a standard protocol, and propofol was not used for sedation in this period. Thus, the effect of nutrition differences between patients was acceptable. Second, inflammatory cytokines, such as TNF-*α*, interleukin-1, and interleukin-6, were not examined in our study. In septic conditions, it is well known that these molecules affect lipid metabolism [35, 36]. These markers may have provided additional information on the relationships between sepsis and lipid profiles. Third, the duration of infection before sepsis was not calculated. Once patients were exposed to LPS by infection, HDL levels decreased, and thus the changes of serum lipid levels over time in this study may have been affected by the duration of time from infection to sepsis in each patient.

In conclusion, the changes in lipid profiles over time differed significantly between the survivor and nonsurvivor groups. Increased serum TG levels may be important in the prognosis of patients with sepsis, as TG levels on days 0 and 1 were significantly associated with 28-day mortality. In addition, the addition of TG to the SOFA score via ROC curve analysis may improve the accuracy of mortality predictions. Large-scale studies are required to confirm the role of lipid and lipoprotein in the outcomes of ICU patients with sepsis.

## Figures and Tables

**Table 1:** Baseline characteristics of patients at ICU admission (*n* = 117, day 0).

Variables	Survivors	Nonsurvivors	*p* value
	(*n* = 65)	(*n* = 52)	
Age, yr	63.0 ± 16.6	62.3 ± 15.9	0.832
Male (%)	42 (64.6)	31 (59.6)	0.579
BMI, kg/m^2^	22.6 ± 4.1	22.9 ± 3.8	0.754
Length of ICU stay, days^*∗*^	11.0 [5, 20.5]	12.5 [3.3, 23.8]	0.695
Severe sepsis	17 (26.2)	2 (3.8)	0.001
Septic shock	48 (73.8)	50 (96.2)	0.001
APACHE II score	18.3 ± 6.0	20.0 ± 5.0	0.082
SOFA score	6.9 ± 3.1	8.6 ± 3.7	0.008
Infection site			0.014
Lung	45 (69.2)	49 (94.2)	
Urinary tract	5 (7.7)	—	
Abdomen	11 (16.9)	3 (5.8)	
Others^*∗*^	4 (6.1)	—	
Underlying diseases			
Diabetes mellitus	21 (32.3)	9 (17.3)	0.065
Chronic lung disease^†^	10 (15.4)	14 (26.9)	0.125
Chronic renal disease	11 (16.9)	9 (17.3)	0.956
Hypertension	27 (41.5)	19 (36.5)	0.582
Heart failure	9 (13.8)	6 (11.5)	0.711
Coronary artery disease	6 (9.2)	6 (11.5)	0.683
Chronic liver disease	4 (6.2)	6 (11.5)	0.301
Clinical parameters			
Hemoglobin, g/dL	10.2 ± 2.2	9.6 ± 2.1	0.151
Platelets, 10^3^/mm^3^	204.9 ± 157.7	177.6 ± 145.6	0.338
Albumin, g/dL	2.7 ± 0.7	2.6 ± 0.6	0.185
Blood urea nitrogen, mg/dL	32.3 ± 27.2	30.9 ± 25.2	0.776
Creatine, mg/dL	2.1 ± 2.1	1.7 ± 1.8	0.224
CRP, mg/dL	15.2 ± 11.1	19.6 ± 14.0	0.068
Lipids^*∗*^			
Cholesterol, mg/dL	106.0 [78.0, 132.0]	84.5 [57.5, 129.5]	0.196
Triglyceride, mg/dL	100.0 [66.5, 188.5]	81.0 [61.8, 113.5]	0.011
High-density lipoprotein, mg/dL	21.0 [13.0, 30.5]	21.0 [9.0, 27.0]	0.411
Low-density lipoprotein, mg/dL	46.0 [29.5, 69.5]	39.0 [22.0, 65.0]	0.414
Free fatty acid, *µ*Eq/L	487.5 [287.0, 742.8]	441.0 [222.0, 570.8]	0.026
Lipoprotein A, mg/dL	7.0 [3.0, 23.1]	6.1 [2.7, 19.0]	0.242

Data are presented as numbers (percentages).

^*∗*^Data are presented as the median [interquartile range].

^†^Chronic lung disease includes asthma, COPD, and structural lung diseases, such as bronchiectasis and interstitial lung disease.

ICU, intensive care unit; BMI, body mass index; APACHE II, Acute Physiology and Chronic Health Evaluation II;* SOFA*, Sequential Organ Failure Assessment.

**Table 2:** Analysis of survival by lipid profiles in ICU patients adjusted for age, sex, body mass index, and Sequential Organ Failure Assessment score.

Variables		Median (IQR)	Hazard ratio	95% CI	*p* value
Day 0	Cholesterol	95.0 [65.5, 132.0]	1.003	0.995 to 1.010	0.469
	TG	92.0 [62.0, 137.3]	0.991	0.984 to 0.998	0.015
	HDL	21.0 [11.3, 28.8]	1.018	0.991 to 1.045	0.192
	LDL	40.0 [26.3, 68.3]	1.008	0.995 to 1.021	0.246
	FFA	457.0 [256.3, 692.0]	0.999	0.998 to 1.000	0.124
	Apo A-I	6.5 [2.8, 19.0]	1.000	0.945 to 1.025	0.986

Day 1	Cholesterol	88.0 [63.8, 116.3]	1.006	0.997 to 1.015	0.210
	TG	93.0 [62.8, 145.3]	0.988	0.980 to 0.995	0.002
	HDL	19.0 [10.0, 26.0]	1.024	0.996 to 1.052	0.091
	LDL	37.0 [22.0, 64.0]	1.015	1.000 to 1.030	0.047
	FFA	313.5 [166.0, 525.0]	0.999	0.997 to 1.000	0.146
	Apo A-I	5.7 [2.7, 17.7]	1.004	0.978 to 1.030	0.780

Day 3	Cholesterol	88.0 [58.0, 113.5]	1.003	0.991 to 1.016	0.606
	TG	90.0 [65.0, 130.0]	0.998	0.991 to 1.004	0.470
	HDL	14.0 [8.0, 26.0]	1.034	0992 to 1.078	0.116
	LDL	42.0 [23.8, 61.3]	1.013	0.994 to 1.032	0.189
	FFA	301.0 [145.0, 394.5]	1.000	0.998 to 1.003	0.649
	Apo A-I	5.3 [2.8, 17.6]	1.006	0.972 to 1.041	0.720

Day 7	Cholesterol	94.0 [76.0, 121.5]	0.994	0.981 to 1.008	0.393
	TG	101.0 [73.0, 136.0]	0.999	0.987 to 1.009	0.728
	HDL	19.5 [13.8, 26.0]	1.032	0.975 to 1.092	0.274
	LDL	47.5 [30.5, 71.5]	0.986	0.968 to 1.005	0.156
	FFA	301.0 [167.5, 403.0]	1.002	1.000 to 1.003	0.112
	Apo A-I	8.5 [2.9, 18.7]	0.980	0.934 to 1.027	0.392

ICU, intensive care unit; TG, triglyceride; HDL, high-density lipoprotein; LDL, low-density lipoprotein; FFA, free fatty acid; Apo A-I, apolipoprotein A-I; IQR, interquartile range.

**Table 3:** Correlations between Sequential Organ Failure Assessment score and the serum concentrations of lipids in ICU patients with sepsis.

	Variable	Correlation (rho)	*p* value
Day 0	Cholesterol	−0.428	<0.001
	TG	−0.028	0.791
	HDL	−0.449	<0.001
	LDL	−0.418	<0.001
	FFA	0.006	0.954
	Apo A-I	−0.279	0.007

Day 1	Cholesterol	−0.454	<0.001
	TG	0.066	0.545
	HDL	−0.470	<0.001
	LDL	−0.49	<0.001
	FFA	0.249	0.021
	Apo A-I	−0.359	0.001

Day 3	Cholesterol	−0.353	<0.001
	TG	0.044	0.712
	HDL	−0.453	<0.001
	LDL	−0.466	<0.001
	FFA	−0.056	0.642
	Apo A-I	−0.414	<0.001

Day 7	Cholesterol	−0.276	0.013
	TG	−0.158	0.231
	HDL	−0.398	0.002
	LDL	−0.312	0.017
	FFA	−0.156	0.248
	Apo A-I	−0.293	0.025

ICU, intensive care unit; TG, triglyceride; HDL, high-density lipoprotein; LDL, low-density lipoprotein; FFA, free fatty acid; Apo A-I, apolipoprotein A-I.
